# Supplementary material for: Tomato Defense against Whiteflies under Drought Stress: Non-Additive Effects and Cultivar-Specific Responses
Source: Plants (Basel). 2022 Apr 12;11(8):1049. doi: 10.3390/plants11081049 (PMC9030952; doi:10.3390/plants11081049)
Supplement: Supplementary file 1 [file plants-11-01049-s001.zip › plants-1628246-supplementary.pdf]

González-Klenner, F.J. Albornoz, M.V., Ávila-Sákar, G., Verdugo, J.A. **Tomato defense against whiteflies under drought stress: Non-additive effects and cultivar-specific responses**

Table S1. Summary statistics of generalized linear model analyses conducted for the study of the effects of drought stress on tomato resistance to whitefly feeding. See methods for details.

**Figure**	**Variable**	**Cultivar**			**Irrigation**			**Whitefly**			**C x I**			**C x W**			**I x W**			**C x I x W**			**n (non-missing)**	**Residual d.f.**	**Statistic**	**Error distrib.**	**SS**
		**d.f.**	**stat**	***P*-value**	**d.f.**	**stat**	***P*-value**	**d.f.**	**stat**	***P*-value**	**d.f.**	**stat**	***P*-value**	**d.f.**	**stat**	***P*-value**	**d.f.**	**stat**	***P*-value**	**d.f.**	**stat**	***P*-value**					
**1**	Type I Trichome density	3	0.918	0.821	1	16.562	<0.0001				3	6.617	0.085										480	472	Chi-sq	Binomial, link = logit	Type I
**2**	Type VI Trichome density	3	240.96	<0.0001	1	1122.91	<0.0001				3	90.11	0.0010										480	472	Chi-sq	Poisson, link=log	Type I
**3**	Type V Non-Gland. Tr. dens.	3	927.2	<0.0001	1	4287.3	<0.0001				3	2271.5	<0.0001										480	472	Chi-sq	Poisson, link=log	Type I
**4**	Whitefly population growth rate	3	12.29	<0.0001	1	22.20	<0.0001				3	20.93	<0.0001										120	112	F	Gaussian, identity	Type I
**5a**	Diameter	3	3.958	0.009	1	7.97	0.005	1	102.71	<0.0001	3	0.165	0.920	3	3.009	0.031	1	0.655	0.419	3	3.186	0.024	256	240	F	Gaussian, identity	Type II
**5b**	Height	3	5.258	0.002	1	71.869	<0.0001	1	102.20	<0.0001	3	2.085	0.103	3	7.395	<0.0001	1	51.060	<0.0001	3	2.948	0.033	256	240	F	Gaussian, identity	Type II
**5c**	Number of Leaves	3	73.14	<0.0001	1	130.15	<0.0001	1	478.09	<0.0001	3	16.32	0.0001	3	37.72	<0.0001	1	42.21	<0.0001	3	31.42	<0.0001	256	240	Chi-sq	Poisson, link=log	Type II
**6**	Veg. Performance (PC1)	3	3.04	0.030	1	23.312	<0.0001	1	156.39	<0.0001	3	0.806	0.492	3	6.985	0.0002	1	22.42	<0.0001	3	3.846	0.010	256	240	F	Gaussian, identity	Type II
**7**	Days to first flower	3	136.00	<0.0001	1	24.59	<0.0001	1			3	50.06	<0.0001										255	247	Chi-sq	Poisson, link=log	Type II
**8a**	Number of fruits	3	32.27	<0.0001	1	89.00	<0.0001	1	173.91	<0.0001	3	2.26	0.520	3	16.26	0.001	1	7.29	0.007	3	2.11	0.551	255	240	Chi-sq	Poisson, link=log	Type II
8b	Fruit fresh weight	3	6.482	0.0003	1	98.88	<0.0001	1	30.35	<0.0001	3	4.772	0.003	3	2.872	0.038	1	0.818	0.367	3	3.978	0.009	208	192	F	Gaussian, identity	Type II
8c	Fruit dry matter	3	3.959	0.009	1	62.71	<0.0001	1	0.114	0.736	3	1.810	0.147	3	1.884	0.137	1	0.562	0.454	3	0.538	0.657	208	192	F	Gaussian, identity	Type II
9	Compensatory ability	3	2.843	0.040	1	8.422	0.004				3	2.893	0.037										160	152	F	Gaussian, identity	Type I

d.f.: degrees of freedom, stat.: statistic (see column for information on statistic used), PC1: Principal Component 1 from Principal Component Analysis
